# RsMYB8-RsMYB73 module positively regulates parthenocarpic fruitsetting via elevating *RsGA3ox9* expression in seedless chestnut rose (*Rosa sterilis*)

**DOI:** 10.1093/hr/uhaf277

**Published:** 2025-10-20

**Authors:** Kui Zhou, Sulin Wen, Yuxin Leng, Silin Zhong, Luonan Shen, Lin Deng, Yi Min, Qiandong Hou, Zhilang Qiu, Yuqing Wang, Lei Peng, Zhenfu Song, Guang Qiao, Xiaopeng Wen

**Affiliations:** Key Laboratory of Plant Resource Conservation and Germplasm Innovation in Mountainous Region (Ministry of Education), Institute of Agro-Bioengineering, College of Life Sciences, Guizhou University, Guiyang 550025, China; Ministry of Agriculture and Rural Affairs Key Laboratory of Crop Genetic Resources and Germplasm Innovation in Karst Region, Guizhou Academy of Agricultural Sciences, Guiyang 550025, China; Key Laboratory of Plant Resource Conservation and Germplasm Innovation in Mountainous Region (Ministry of Education), Institute of Agro-Bioengineering, College of Life Sciences, Guizhou University, Guiyang 550025, China; Key Laboratory of Plant Resource Conservation and Germplasm Innovation in Mountainous Region (Ministry of Education), Institute of Agro-Bioengineering, College of Life Sciences, Guizhou University, Guiyang 550025, China; Anshun Academy of Agricultural Sciences, Anshun 562100, China; Key Laboratory of Plant Resource Conservation and Germplasm Innovation in Mountainous Region (Ministry of Education), Institute of Agro-Bioengineering, College of Life Sciences, Guizhou University, Guiyang 550025, China; Key Laboratory of Plant Resource Conservation and Germplasm Innovation in Mountainous Region (Ministry of Education), Institute of Agro-Bioengineering, College of Life Sciences, Guizhou University, Guiyang 550025, China; Key Laboratory of Plant Resource Conservation and Germplasm Innovation in Mountainous Region (Ministry of Education), Institute of Agro-Bioengineering, College of Life Sciences, Guizhou University, Guiyang 550025, China; Key Laboratory of Plant Resource Conservation and Germplasm Innovation in Mountainous Region (Ministry of Education), Institute of Agro-Bioengineering, College of Life Sciences, Guizhou University, Guiyang 550025, China; Key Laboratory of Environmental Pollution Monitoring and Disease Control of Ministry of Education, Immune Cells and Antibody Engineering Research Center of Guizhou Province, School of Biology and Engineering (School of Health Medicine Modern Industry), Guizhou Medical University, Guiyang 550025, China; Key Laboratory of Plant Resource Conservation and Germplasm Innovation in Mountainous Region (Ministry of Education), Institute of Agro-Bioengineering, College of Life Sciences, Guizhou University, Guiyang 550025, China; Key Laboratory of Plant Resource Conservation and Germplasm Innovation in Mountainous Region (Ministry of Education), Institute of Agro-Bioengineering, College of Life Sciences, Guizhou University, Guiyang 550025, China; Anshun Academy of Agricultural Sciences, Anshun 562100, China; Key Laboratory of Plant Resource Conservation and Germplasm Innovation in Mountainous Region (Ministry of Education), Institute of Agro-Bioengineering, College of Life Sciences, Guizhou University, Guiyang 550025, China; Key Laboratory of Plant Resource Conservation and Germplasm Innovation in Mountainous Region (Ministry of Education), Institute of Agro-Bioengineering, College of Life Sciences, Guizhou University, Guiyang 550025, China

## Abstract

Fruit growth and development are generally initiated following successful pollination and fertilization. Seedless chestnut rose (*Rosa sterilis*), an elite promising fruit tree for both edible and medicinal purposes due to the extremely high vitamin C and superior quality, exhibits a naturally parthenocarpic character, however the underlying mechanism has been still unclear so far. Currently, gibberellins (GAs) were justified as the key hormone for parthenocarpy induction in seedless chestnut rose by endogenous hormone analysis and exogenous plant growth regulator (PGR) application. In total, 43 members of the GA oxidase gene family (*RsGAoxs*) were systematically identified and characterized based on genome-wide analysis of seedless chestnut rose. On the basis of transcriptomic analysis, overexpression experiments in tomato, as well as virus-induced gene silencing (VIGS) assay in seedless chestnut rose, *RsGA3ox9* was substantially justified to be involved in the parthenocarpic fruitsetting of this species. Transcription factors RsMYB3, RsMYB8, and RsMYB73 were proven to positively regulate the expression of *RsGA3ox9*. Further, yeast two-hybrid (Y2H) and luciferase complementation assay illuminated that RsMYB8 and RsMYB73 may interact, leading to upregulating *RsGA3ox9*. Thereby, *RsGA3ox9* substantially regulates parthenocarpy of seedless chestnut rose, and RsMYB8-RsMYB73 complex promotes parthenocarpic fruitsetting by upregulating *RsGA3ox9*, which may facilitate the seedless fruit breeding in chestnut rose (*Rosa roxburghii* Tratt.), as well as provide novel insights for better understanding the mechanism underlying the parthenocarpic fruitsetting in fruit species.

## Introduction

In angiosperms, pollination and fertilization constitute a necessary step for fruitsetting. Generally, if these processes encounter obstacles, it may cause ovule abortion, followed by ovary abscission, and ultimately fails to develop into fruits [1]. However, in some species, e.g. tomatoes, seedless watermelons, grapes, and pears, etc., fruitlets may growth and develop without undergoing pollination and fertilization [2]. Under conditions of severe pollen abortion or absence of pollination, their ovaries can still develop into fruits, which is termed as parthenocarpy. Parthenocarpy represents a critical pathway for inducing seedless fruit. In fruit production, parthenocarpic fruit not only demonstrate better fruit quality and superior edible rate, but are also highly favored by consumers, thereby holding significant practical utility and commercial value [3].

Parthenocarpy can be categorized into natural parthenocarpy and stimulative parthenocarpy. Natural parthenocarpy refers to the phenomenon where ovaries or receptacles develop directly into seedless fruits without any external stimulation or treatment, and stimulative parthenocarpy describes the development of ovaries into fruits under external stimuli such as pollen, plant growth regulators (PGRs), or environmental factors. However, the genetic laws of natural parthenocarpy and its underlying genetic mechanisms remain unclear [4]. Unlike natural parthenocarpy, stimulative parthenocarpy is nonheritable and requires repeated stimulation to induce seedless fruit production [5].

Many factors, such as chromosomal ploidy abnormalities, male sterility, fertilization disorders, abnormal morphological structure of flower organs, hormone induction etc., may lead to parthenocarpy [6, 7]. Among the plant hormones, auxin (IAA), gibberellins (GAs), cytokinins (CTKs), ethylene (ETH), as well as brassinosteroids (BRs), etc., may induce parthenocarpy, which were documented in many crops [8]. Many hormones, e.g. IAA [9], GAs [10], and ETH [11], were justified to induce parthenocarpy in tomato. In pear, spraying of 2,4-dichlorophenoxyacetic acid (2,4-D) induces higher GA content, promotes fruit division and differentiation, and ultimately leads to parthenocarpy fruit production [12]. The exogenous GA_4 + 7_ treatment promoted pear fruitsetting without pollination, while the GA_1 + 3_ did not produce parthenocarpy [13]. In grape, spraying PGRs, e.g. GA_3_, 4-chlorophenoxyacetic acid (4-CPA), 6-benzylaminopurine (6-BA), induce parthenocarpic fruit [14].

To date, function genes involving in parthenocarpy had been reported in several plant species. In pear, the overexpression of the gene *PbGA20ox2* increased GA_4_ content, and delayed the shedding of the unpollinated ovary. Interestingly, the stable transfer of *PbGA20ox2* into tomato induced the unpollinated ovaries into fruits, which ultimately led to the production of parthenocarpy [15]. In *Solanaceae* plants such as eggplant, pepper, and tomato, silencing the *PAD-1* gene leads to higher IAA content in the unpollinated ovary, resulting in parthenocarpy [16]. In cucumber, IAA signaling genes *AUX22A-like1*, *AUX22B-like2*, and *AUX28-like* were expressed at higher levels in parthenocarpic DDX lines than in nonparthenocarpic ZK lines [17]. In addition, tomato with RNAi of the gene *SlGA2ox* exhibited high levels of active GA_1_ and GA_4_ in stems and fruits, which ultimately gave rise to parthenocarpy [18].

Seedless chestnut rose (*Rosa sterilis* S. D. Shi), also named as ‘seedless Cili’, is characterized by extremely high vitamin C, superior soluble solids content (SSC). and bearing seedless fruit [19], and is recognized as an independent relative of chestnut rose (*Rosa roxburghii* Tratt.). As an elite germplasm of Guizhou Province, China, seedless chestnut rose is labeled as a promising third-generation fruit with distinguished potential in exploitation [20]. In 2004, Wen *et al*. proposed that it was probably derived from a mutant of severe male-sterile *Rosa kweichowensis* by RAPD and AFLP analyses [21]. Recently, Zong *et al*. suggested that it originated from the hybridization between *R. roxburghii* and *Rosa longicuspis* based on genomic analysis [22]. Due to the absence of seeds, seedless chestnut rose is mainly propagated by means of cutting and grafting. In our previous study, the morphological and cytological observations demonstrated that severe male sterility existed in seedless chestnut rose since a small amount of pollen grains were observed from the anthers, majority of which were nonviable [19]. Despite pollen abortion, successful fruitsetting is nevertheless achieved, yet the mechanism underlying this phenomenon remains unclear.

Parthenocarpy of seedless chestnut rose is a rare phenomenon among the *Rosa*, elucidating the molecular mechanism underlying the fruitsetting of this fruit crop may provide an important insight for the seedless fruit breeding of chestnut rose. The fruit development of seedless chestnut rose reflects its parthenocarpy ability, and investigating fruit development is the most direct way to unravel the mechanism of parthenocarpy. Currently, the key hormones for parthenocarpic fruitsetting were investigated via endogenous hormone analysis and PGR application during fruit growth and development stages in seedless chestnut rose. Genome-wide identification of *RsGAoxs* was performed; subsequently, transcriptomic profiling, overexpression in tomato, and VIGS experiments in seedless chestnut rose were also carried out to justify the role of *RsGA3ox9* in regulating parthenocarpy. The promoter region of *RsGA3ox9* was cloned and subjected to yeast one-hybrid (Y1H) screening so as to identify transcription factors (TFs) regulating its expression. Further, yeast two-hybrid (Y2H) and luciferase complementation assay were employed to ultimately identify a molecular module regulating parthenocarpy in this fruit crops. This research provides novel insights into the mechanism of parthenocarpy in *Rosa* plants, which may considerably facilitate the seedless fruit breeding in chestnut rose fruits.

## Results

### Dynamics of endogenous hormones during the fruit growth and development of seedless chestnut rose

**Figure 1:**
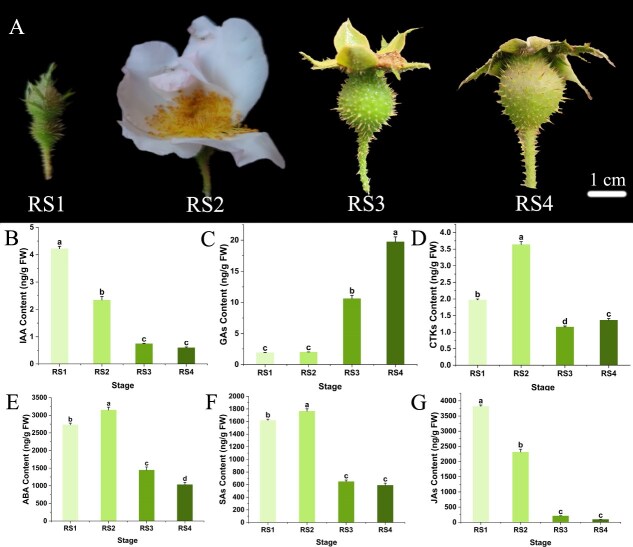
Morphological characters and hormone contents during four fruit stages in seedless chestnut rose. (A) Ovary/fruit in four developmental stages. (B)–(G) The contents of IAA, GAs, CTKs, ABA, SAs and JAs, consecutively. RS1–RS4 indicate the ovary at the seventh day before flowering, the ovary at full flowering, the fruit at the 14th day after flowering, and the fruit at the 28th day after flowering, consecutively. Data with three biological replicates were given as mean values ± standard deviation (SD), different letters within same panel indicated significant difference (*P* < 0.05).

During the fruit growth and development of seedless chestnut rose, remarkable changes in morphological features occur. Starting from RS1 (7th day before flowering) to RS4 (28th day after flowering), the fruits continuously enlarge, and the color shifts from dark green to pale green (Fig. 1A). Samples from RS1 to RS4 were collected to investigate the dynamic alterations of endogenous hormones. The results indicated that with the development of fruits, the contents of IAA and jasmonates (JAs) decreased gradually, and remained at low level in stages RS3 and RS4 (Fig. 1B, G). CTKs, abscisic acid (ABA), and salicylic acids (SAs) initially increased, peaked at the full flowering stage, and then declined (Fig. 1D–F). GAs was extremely low in the initial two stages, but increased significantly from the fruitsetting stage (Fig. 1C), suggesting the involvement of GAs in fruit growth and development of seedless chestnut rose.

### Gibberellins promote parthenocarpy in seedless chestnut rose

**Figure 2:**
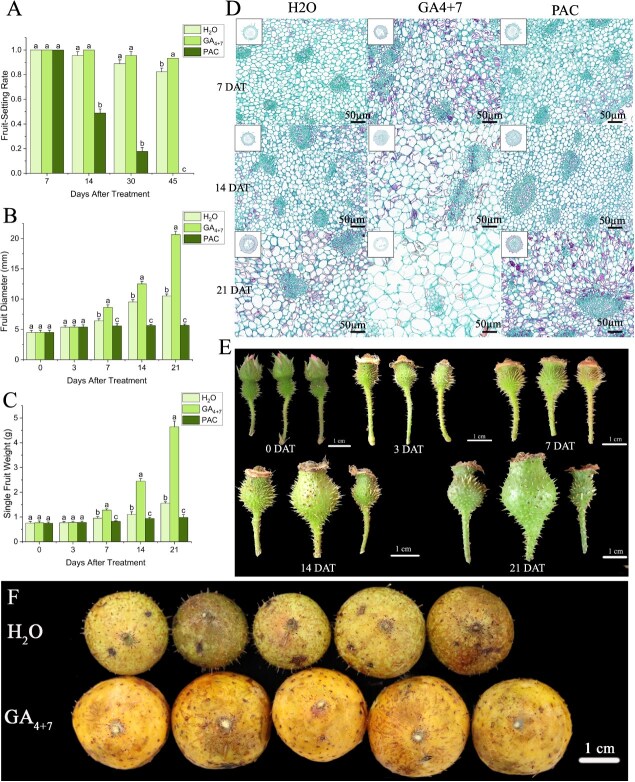
Effects of GA on the fruitsetting rate and fruit development in seedless chestnut rose. (A) The fruitsetting rate as treated with H_2_O, GA_4 + 7_, and PAC. (B) The fruit diameter. (C) The weight per fruit. (D) The cell sizes of the fruits. (E) The fruit features as treated with H_2_O (left), GA_4 + 7_ (middle), and PAC (right). (F) The mature fruits under the treatment of H_2_O and GA_4 + 7_. Data with three biological replicates were given as mean values ± SD, different letters within same panel indicated significant difference (*P* < 0.05).

To further investigate the relationship between GAs and fruitsetting in seedless chestnut rose, the flowers were emasculated before flowering, and then GA_4 + 7_, paclobutrazol (a GA synthesis inhibitor, PAC), and H_2_O (control) were sprayed to observed their effects on fruitsetting and development. The results showed that on the 14th day after treatment (DAT), the fruitlets treated with PAC began to drop, and the fruitsetting rate decreased to 48.88%. On 30 DAT, the fruitsetting rate of GA_4 + 7_, PAC, and H_2_O were 95.56%, 17.78%, and 88.89%, respectively, and the fruitsetting rate under the PAC treatment dropped sharply. On 45 DAT, the fruitsetting rate under GA_4 + 7_ and H_2_O sprayed were as high as 93.33% and 82.22%, respectively; however, all the fruits dropped off as treated by PAC (Fig. 2A). Therefore, seedless chestnut rose highly characterizes in naturally parthenocarpic ability, and GAs play an important role in fruitsetting.

From 7 DAT to 21 DAT, fruit and cell size were observed across the various treatments. Fruits treated with GA_4 + 7_ exhibited a significantly greater diameter and weight compared to those treated with H_2_O, while treatment with H_2_O surpassed that with PAC. Also, fruits treated with both GA_4+7_ and H_2_O continued to enlarge, whereas PAC-treated fruits plateaued in size by 7 DAT and ceased further growth prior to abscission (Fig. 2B, C, E). Additionally, significant differences in cell size were observed among the treatments. At the same tested stages, treatment with GA_4 + 7_ gave rise to larger cell sizes (Fig. 2D), leading to the bigger fruits despite that no significant cell division was investigated. In contrast, fruits treated by PAC exhibited smaller cell sizes than under the GA_4 + 7_ and H_2_O treatment (Fig. 2D). At the fruit ripening stage, fruits treated with GA_4 + 7_ ripened earlier and were significantly larger than those treated with H_2_O (Fig. 2F; Table S1). Collectively, GAs also give an important contribution to fruit development in seedless chestnut rose.

### Key genes involved in parthenocarpic fruitsetting in seedless chestnut rose

**Figure 3:**
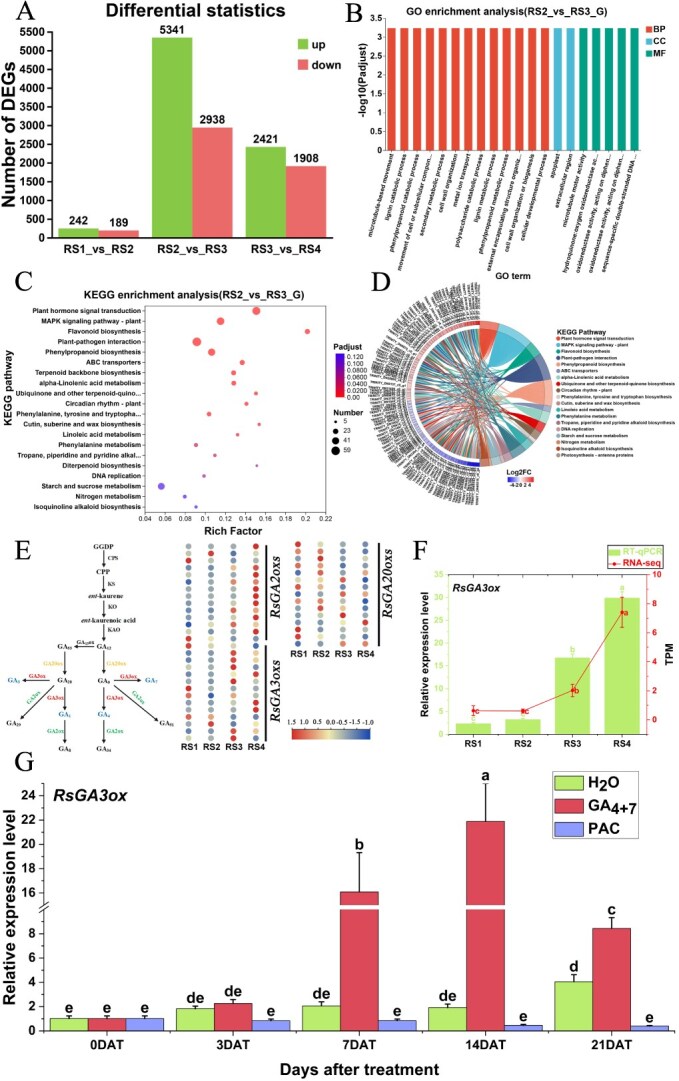
Enrichment analysis of DEGs and expression patterns of the gene *RsGA3ox* in seedless chestnut rose. (A) Statistics of DEGs during four stages. (B) GO enrichment analysis of DEGs between RS2 and RS3. (C) KEGG enrichment analysis of DEGs between RS2 and RS3. (D) KEGG enrichment chord diagram analysis of DEGs between RS2 and RS3. (E) Gibberellin biosynthesis pathway and the transcriptome expression heatmap of the corresponding genes. (F) Relative expression levels of *RsGA3ox* during four stages. (G) The expression patterns of *RsGA3ox* under treatments with H_2_O, GA_4 + 7_, and PAC. Data with three biological replicates were given as mean values ± SD, different letters within same panel indicated significant difference (*P* < 0.05).

A total of 113 310 genes were identified through transcriptome sequencing across the four developmental stages of seedless chestnut rose fruits. Significance analysis of adjacent differentially expressed genes (DEGs) indicated that the highest number of DEGs, totaling 8279, was observed between the full flowering stage (RS2) and the early fruitsetting stage (RS3), which was followed by 4329 DEGs between RS3 and RS4, while the least number of DEGs, only 431, was found between RS1 and RS2 (Fig. 3A). Gene ontology (GO) and Kyoto encyclopedia of genes and genomes (KEGG) enrichment analyses were performed on the DEGs between the full flowering stage (RS2) and the early fruitsetting stage (RS3). Among the top 20 significant terms identified through GO enrichment, the majority were classified under biological processes (BP). These DEGs were primarily involved in biosynthesis and decomposition processes, cell wall organization, and cellular developmental processes, which are closely associated with fruitsetting (Fig. 3B).

KEGG enrichment analysis revealed that the DEGs at the full flowering and early fruitsetting stages were predominantly concentrated on the pathways related to flavonoid biosynthesis, diterpenoid biosynthesis, and plant hormone signal transduction (Fig. 3C). Additionally, an enrichment chord diagram analysis was conducted on the top 20 KEGG pathways. The results indicated that most genes were distributed across distinct pathways and participated in various biological processes. For example, the gene *TRINITY_DN5137_c0_g1* (aromatic amino acid aminotransferase) was found to be involved in multiple pathways, including isoquinoline alkaloid biosynthesis, trapeptide, piperidine, and pyridine alkaloid biosynthesis, phenylalanine metabolism, and the biosynthesis of quinones and other terpenoid–quinone derivatives (Fig. 3D). In this study, diterpenoid biosynthesis was analyzed in depth to elucidate the expression patterns of related genes within the GA biosynthesis pathway.

KEGG enrichment analysis of the RS2 and RS3 stage revealed that the gene *RsGA3ox* (transcriptome ID: TRINITY_DN12053_c0_g2; GA biosynthesis pathway ID: 1.14.11.15) was significantly upregulated in the GA_1_ and GA_4_ biosynthesis pathways, which conformed with the fluctuation in GA contents from the full flowering stage to early fruitsetting stage, indicating the potential role of *RsGA3ox* in GAs biosynthesis (Fig. 3E). Also, the expression pattern of *RsGA3ox* was quantified across the four stages, and no significant change was investigated from RS1 and RS2. Interestingly, *RsGA3ox* was significantly upregulated from RS2 to RS4 (Fig. 3F). Subsequently, the expression patterns of *RsGA3ox* under treatments with H_2_O, GA_4 + 7_, and PAC was analyzed. The results showed that *RsGA3ox* was upregulated on 7 DAT and continued until 14 DAT under treatment with GA_4 + 7_, by contrast, its expression level remained at a very low level under PAC treatment (Fig. 3G), reflecting the strong function of *RsGA3ox* in both fruit growth and development in this fruit species.

### Gibberellin oxidase genes identified from seedless chestnut rose

**Figure 4:**
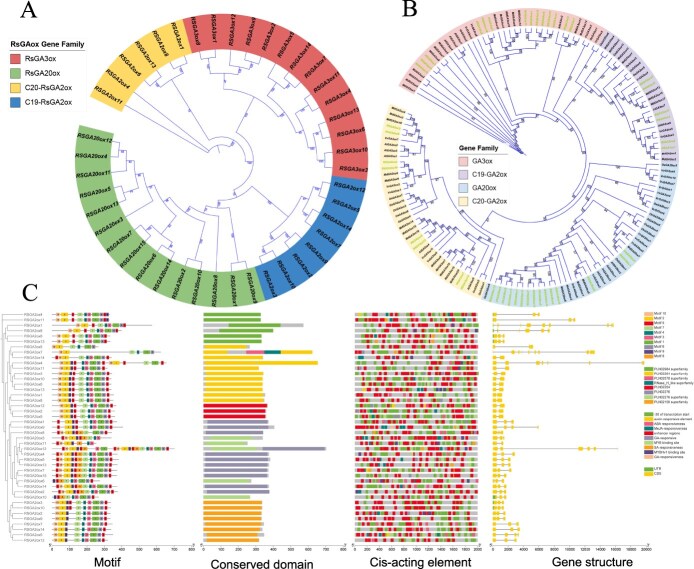
Genome-wide identification of gibberelli oxidase gene (*GAoxs*) family in seedless chestnut rose. (A) Identification of *GAoxs* family members. (B) Phylogenetic tree of *RsGAoxs* with *GAoxs* genes from *A. thaliana*, *O. sativa*, *V. vinifera*, and *M. domestica*. (c) Analysis of motifs, conserved domains, *cis*-acting elements, and gene structures of *RsGAoxs*.

To elucidate the genes critical for fruit growth and development, members of the gibberellin oxidase gene (*GAoxs*) family were investigated via genome-wide analysis of seedless chestnut rose. In total, 43 members of *GAoxs* family were identified from seedless chestnut rose, which may be classified into three subfamilies: 14 *RsGA2ox* (*Gibberellin 2-oxidase*) members, 14 *RsGA3ox* (*Gibberellin 3-oxidase*) members, and 15 *RsGA20ox* (*Gibberellin 20-oxidase*) members (Fig. 4A). Based on their chromosomal positions, these genes were designated as *RsGA2ox1* to *RsGA2ox14*, *RsGA3ox1* to *RsGA3ox14*, and *RsGA20ox1* to *RsGA20ox15*, respectively. Chromosomal distribution analysis revealed that *RsGAoxs* were located on 13 of the 14 chromosomes, with chromosome A5 being the only exception. Chromosomes A7 and B7 harbored the highest number of *RsGAoxs* (Fig. S1). A phylogenetic tree was constructed using the 43 identified *GAoxs* from the seedless chestnut rose, along with those from *Arabidopsis thaliana*, *Oryza sativa*, *Vitis vinifera*, and *Malus domestica*. It was demonstrated that all members were clustered into distinct subfamilies, and the number of *GAoxs* in the seedless chestnut rose is close to apple and significantly higher than those from the other three species examined, suggesting that the seedless chestnut rose and apple possess greater potential for GA biosynthesis and metabolism compared to the other species (Fig. 4B). Subsequently, the seven important indicators, e.g. number of amino acids, molecular weight, isoelectric point, instability index, aliphatic index, hydrophilicity, and subcellular localization were predicted for the 43 *RsGAoxs* (Table S2). This information provides critical insights into the molecular characteristics of the 43 genes, and provides a theoretical basis for subsequent studies.

**Figure 5:**
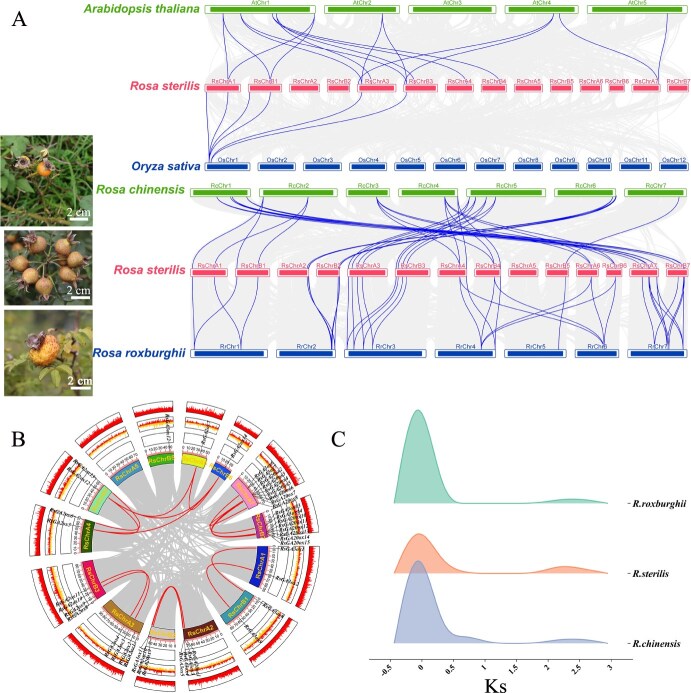
Collinearity analysis of *GAsox* family in seedless chestnut rose. (A) Genome-wide collinearity analysis of *RsGAoxs* with *A. thaliana*, *O. sativa*, *R. chinensis*, and *R. roxburghii*. (B) Self-collinearity analysis of *RsGAoxs*. (C) Synonymous substitution rate (*Ks*) of *GAoxs* distribution among chestnut rose (*R. roxburghii*), seedless chestnut rose (*R. sterilis*), and *R. chinensis*.

**Figure 6:**
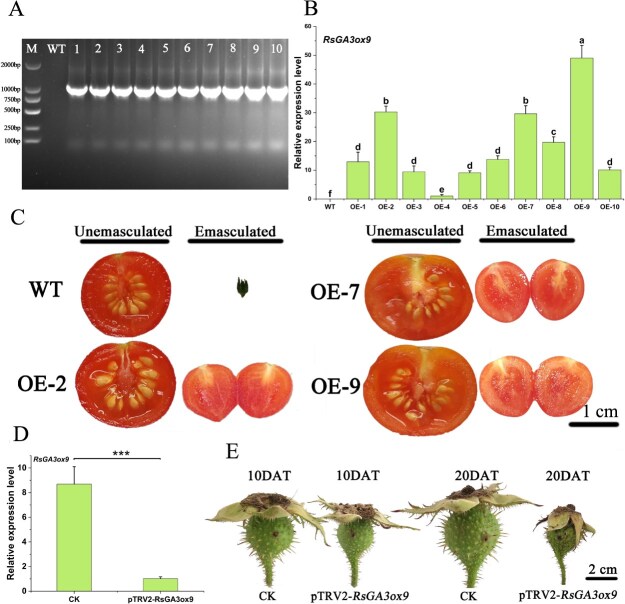
The function of *RsGA3ox9* was collectively validated through overexpression in tomato and VIGS in seedless chestnut rose fruits. (A) PCR validation of transgenic plants. (B) Relative expression levels of *RsGA3ox9* in WT and the overexpressing lines, the relative expression level of OE-4 was set to 1. (C) Fruit phenotypes of the WT and the overexpressing lines under unemasculated and emasculated treatments. (D) Relative expression levels of *RsGA3ox9* in control (CK) and silenced (VIGS) fruits. (E) Fruit phenotypes of the control and the silenced on 10 and 20 DAT. Data with three biological replicates were given as mean values ± SD, different letters within same panel indicated significant difference (*P* < 0.05), asterisk indicates significant difference (Student’s *t*-test, ^***^P < 0.001).

Gene structure analysis of the 43 members revealed that the majority of genes lacked 5′ untranslated regions (UTRs), with coding sequences (CDS) primarily ranging within 1500 bp. Conserved domain analysis indicated that each major *RsGAox* subfamily contained specific conserved domains: *RsGA2ox* and *RsGA20ox* each possess two specific conserved domains, while *RsGA3ox* contains four specific conserved domains. *Cis*-acting element analysis demonstrated that within the 2000-bp upstream regions of these genes, there are several elements, including GA-responsive elements, MYB-binding sites, auxin-responsive elements, and abscisic acid-responsiveness elements, which are closely associated with hormone responses (Fig. 4C). Meanwhile, the expression patterns (TPM) of the 43 *RsGAoxs* were analyzed, and the results revealed that *RsGA2oxs* genes exhibited low expression levels during early fruitsetting stages but higher expression in later stages (Fig. S2). In contrast, the expression levels of *RsGA3oxs* and *RsGA20oxs* were high in the early stage and decreased in the late stage. This indicates that *RsGA3oxs* and *RsGA20oxs* are consistent with the fruit developmental trend and may contribute to fruit development in seedless chestnut rose fruit.

To investigate the collinearity of *RsGAoxs*, both interspecific and intraspecific collinearity analyses were performed based on genomic data. The results indicated that *O. sativa* exhibited the fewest collinear gene pairs with seedless chestnut rose, comprising only six pairs located on chromosome 1 of *O. sativa*. In contrast, *A. thaliana* shared 16 collinear gene pairs with seedless chestnut rose, distributed across chromosomes 1, 2, 4, and 5, with the highest concentration found on chromosome 1 (Fig. 5A, top panel). *Rosa chinensis* and *R. roxburghii* demonstrated more extensive collinear relationships with seedless chestnut rose, exhibiting 37 and 39 pairs, respectively. Notably, *R. roxburghii* displayed the closest collinearity, with the chromosomal positions and orders of collinear genes being highly conserved (Fig. 5A, lower panel).

Subsequent analyses of self-collinearity in seedless chestnut rose revealed that 30 genes exhibited collinear relationships, most of which resulted from duplication events between chromosomes in groups A and B. Notably, the collinearity of *RsGA2ox5*, *RsGA2ox6*, *RsGA2ox7*, and *RsGA2ox14* occurred specifically between chromosomes 4 and 6, which was plausibly ascribed to gene amplification events (Fig. 5B). Concurrently, we calculated the synonymous substitution rates (*Ks*) of *RsGAoxs* among *R. roxburghii*, seedless chestnut rose (*R. sterilis*), and *R. chinensis* to model whole-genome duplication (WGD) events across these three species. The results revealed a prominent peak from −0.5 to 0.5, indicating a significant gene duplication event shared by all three species, potentially coinciding with the ancestral eudicot radiation. Additionally, a minor peak from 2 to 2.5 exhibited a slight temporal lag compared to previously reported WGD timelines based on whole-genome analyses [23], suggesting a delayed duplication timing of *RsGAoxs* in the genus *Rosa* (Fig. 5C).

### The role of *RsGA3ox9* in regulating parthenocarpy of seedless chestnut rose

To identify the specific member of the *RsGA3ox* family, we performed a sequence alignment, and *RsGA3ox9* was identified, which exhibited 100% sequence identity with the former candidate gene, *RsGA3ox*. To validate the association between *RsGA3ox9* and parthenocarpy, we conducted functional analyses through overexpression in tomato and VIGS assays in seedless chestnut rose. The CaMV35S promoter was used, following selection, differentiation, and rooting processes, complete transgenic plants were regenerated (Fig. S2A–D). A total of 10 transgenic lines were obtained (OE-1 to OE-10, indicating overexpression of tomato lines 1–10, consecutively) (Fig. 6A), with the three lines (OE-2, OE-7, and OE-9) exhibiting very high expression levels of *RsGA3ox9* selected for further analyses (Fig. 6B). Two days prior to flowering, the flowers of both transgenic plants and wild type (WT) were emasculated for phenotypic observation (Fig. S2E). The results demonstrated that under unemasculated treatment, both transgenic and WT plants developed fruits normally, and with no significant difference of phenotype (Table 1). In contrast, under emasculated treatment, the ovaries dropped rapidly without fruit formation in WT, while the ovaries continued to develop seedless fruits normally in transgenic plants (Fig. 6C). However, transgenic plants with the emasculation exhibited a lower fruitsetting rate (average of 10.2%–17.3%) and smaller fruit weights (average of 1.25–1.27 g) compared to the unemasculated controls (Table 1).

To directly validate the role of *RsGA3ox9* in parthenocarpy, VIGS assays were also performed on fruits of seedless chestnut rose. The results revealed a significant reduction *RsGA3ox9* expression from the silenced fruits compared to the control on 10 DAT, confirming the effectiveness of gene silencing (Fig. 6D). Furthermore, the silenced fruits displayed the stunted growth, with their diameter smaller than those of the control. On 20 DAT, the fruits had completely withered and eventually abscised by VIGS (Fig. 6E). It’s revealed that *RsGA3ox9* highly contributes to the fruit development of seedless chestnut rose.

### Positive involvement of RsMYBs in parthenocarpy via regulating *RsGA3ox9*

**Figure 7:**
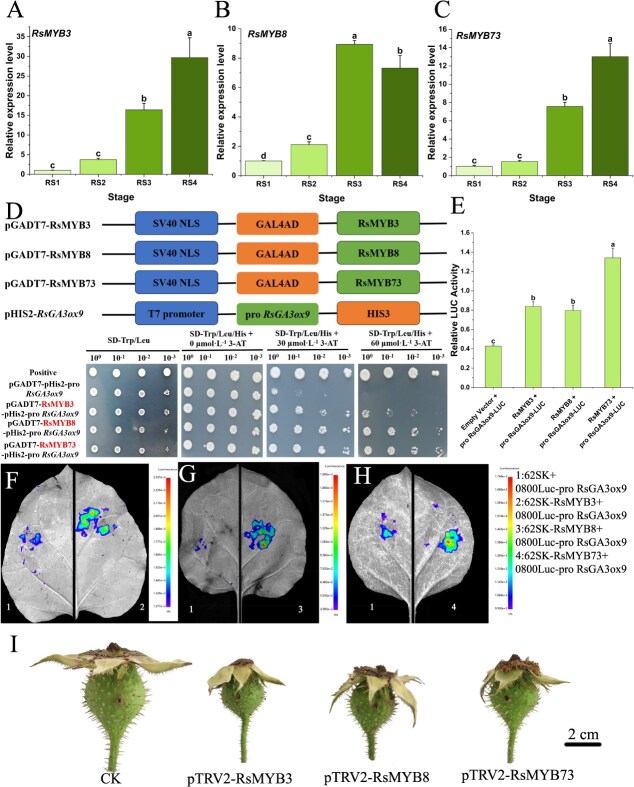
RsMYBs contribute to parthenocarpic fruitsetting by positively regulating *RsGA3ox9* expression in seedless chestnut rose. (A)–(C) Relative expression analysis of RsMYB3, RsMYB8, and RsMYB73 across four developmental stages, consecutively. (D) Y1H assay showing interaction between RsMYB3/RsMYB8/RsMYB73 and *RsGA3ox9* promoter. (E) Firefly luciferase/Renilla luciferase ratio analysis. (F)–(H) Dual-luciferase assay results for RsMYB3/RsMYB8/RsMYB73 and *RsGA3ox9* promoter interaction, consecutively. (I) Fruit phenotypes of the control and the silenced by VIGS. Data with three biological replicates were given as mean values ± SD, different letters within same panel indicated significant difference (*P* < 0.05).

*Cis*-acting element analysis of the *RsGA3ox9* promoter revealed MYB-binding sites within the 2000-bp upstream regulatory region (Fig. 4C). Based on this finding, 33 members of the MYB TF family were identified from the transcriptome, and the TPM (Transcripts Per Million) levels were statistically analyzed for each TF (Fig. S3A). According to this result, RsMYB3, RsMYB8, and RsMYB73 were selected for quantitative real-time polymerase chain reaction (RT-qPCR) analyses during fruit development stages of seedless chestnut rose, which exhibited significant differential expression between RS2 and RS3 (Fig. 7A–C), and were similar to the expression pattern of gene *RsGA3ox9*. Subcellular localization assays results showed that all three TFs localized to the nucleus (Fig. S3B). Next, recombinant plasmids pHIS2-*pro RsGA3ox9*, pGADT7-RsMYB3, pGADT7-RsMYB8, and pGADT7-RsMYB73 were constructed and cotransformed into yeast strain Y187. The results demonstrated that RsMYB3, RsMYB8, and RsMYB73 all bound to the *RsGA3ox9* promoter, suggesting that these TFs may regulate *RsGA3ox9* expression (Fig. 7D).

Subsequently, dual-luciferase reporter assays were performed to investigate the regulatory roles of the three TFs in *RsGA3ox9*. The results indicated that RsMYB3, RsMYB8, and RsMYB73 all positively regulated *RsGA3ox9* expression, which was consistent with their corresponding expression patterns (Fig. 7E–H). Finally, VIGS assays was carried out for the three TFs in seedless chestnut rose fruit. After confirming their silence efficiency (Fig. S4A–C), fruit size was investigated on 10 DAT. It was revealed that silenced fruits were significantly smaller than the control (Fig. 7I). Therefore, RsMYB3, RsMYB8, and RsMYB73 substantially contribute to parthenocarpic fruitsetting in seedless chestnut rose by positively regulating *RsGA3ox9* expression.

### Cooperative regulation of *RsGA3ox9* by RsMYB8–RsMYB73 complex

**Figure 8:**
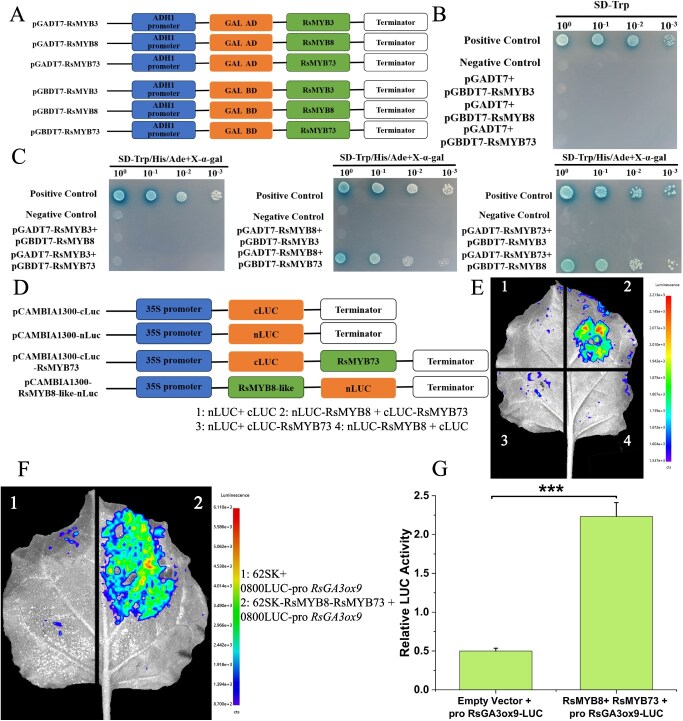
RsMYB8–RsMYB73 complex positively regulation of *RsGA3ox9*. (A) Pattern diagram of RsMYB3, RsMYB8, and RsMYB73 constructed into vectors pGADT7 and pGBKT7. (B) Self-activation validation of RsMYB3, RsMYB8, and RsMYB73. (C) Y2H assays for interactions among three TFs. (D) Pattern diagram of RsMYB8 and RsMYB73 constructed into vectors nLuc and cLuc, respectively. (E) Luciferase complementation assays for RsMYB8 and RsMYB73. (F) Dual-luciferase reporter assays of RsMYB8 and RsMYB73 coinfiltrated into tobacco. (G) Firefly luciferase/Renilla luciferase ratio analysis. Data with three biological replicates were given as mean values ± SD, asterisk indicates significant difference (Student’s *t*-test, ^***^P < 0.001).

After confirming that the three TFs positively regulate *RsGA3ox9*, we further investigated whether they act cooperatively or antagonistically. Recombinant plasmids, pGBKT7-RsMYB3, pGBKT7-RsMYB8, and pGBKT7-RsMYB73 were constructed and spotted onto SD-Trp-His-Ade solid medium. The results indicated that none of the three TFs exhibited self-activation activity (Fig. 8B). Based on this, six groups of Y2H assays were carried out to examine the interactions between every TF and the other two. It was shown that only combinations pGADT7-RsMYB8 + pGBKT7-RsMYB73 and pGADT7-RsMYB73 + pGBKT7-RsMYB8 turned blue on SD-Trp-Leu-His-Ade solid medium with X-α-gal, indicating an interaction between RsMYB8 and RsMYB73, the complex for RsMYB8–RsMYB73 may cooperatively regulate the expression of *RsGA3ox9*.

**Table 1:** Parthenocarpic capacity of transgenic tomato and the WT

	**Unemasculated**		**Emasculated**	
**Lines**	**Fruitsetting rate**	**Fruit weight (g)**	**Fruitsetting rate**	**Fruit weight (g)**
WT	0.984 ± 0.011^a^	4.92 ± 0.07^a^	0^b^	0^c^
OE-2	0.993 ± 0.010^a^	4.91 ± 0.07^a^	0.155 ± 0.019^a^	1.25 ± 0.08^a^
OE-7	0.985 ± 0.011^a^	4.94 ± 0.07^a^	0.102 ± 0.019^c^	1.38 ± 0.16^a^
OE-9	0.984 ± 0.011^a^	4.95 ± 0.07^a^	0.173 ± 0.008^a^	1.47 ± 0.17^a^

Data with three biological replicates were given as mean values ± SD, values marked with different letter within a column stand for significance at *P* < 0.05.

Subsequently, the interaction between RsMYB8 and RsMYB73 was further validated. Recombinant plasmids nLuc-RsMYB8 and cLuc-RsMYB73 were successfully constructed, transformed into *Agrobacterium*, and infiltrated into tobacco leaves. The results showed that the experimental group (nLuc-RsMYB8 + cLuc-RsMYB73) emitted high fluorescence, further indicating that the two molecules could interact with each other (Fig. 8E). Additionally, dual-luciferase reporter assays were performed with the two TFs and *RsGA3ox9*, to determine whether their complex would regulate the expression of *RsGA3ox9*. It was demonstrated that coinfiltration of RsMYB8 and RsMYB73 into tobacco resulted in significantly higher Firefly luciferase/Renilla luciferase ratios (Figs 7D and 8F, G), indicating the positive regulation of RsMYB8–RsMYB73 in *RsGA3ox9* expression.

## Discussion

### GA is the key hormone for parthenocarpy of seedless chestnut rose

In fruit trees, parthenocarpy is a favorite agronomic trait that not only improves fruit quality and edibility but also produces seedless fruits favored by consumers, offering significant application and economic value in agricultural production [16]. Recent advancements in horticultural crops, including pear, cucumber, banana, watermelon, grape, and tomato, etc., have led to the genesis of parthenocarpic lines through hormonal induction or genetic modification, thus broadening consumer choices with superior fruit varieties [8]. Despite the substantial market demand in this area, the regulatory mechanism underlying the parthenocarpy in horticultural crops remains poorly understood, especially in higher plants like trees and shrubs. In some crops, external factors such as temperature, light, vibration, and the application of PGRs, etc., can induce parthenocarpy. Among these, the use of PGRs is more prevalent in actual production [14].

Hormonal treatments, e.g. IAA, GAs, CTK, and ETH, can induce parthenocarpic fruit development. Available evidences have shown that, within the same species, ovaries of parthenocarpic plants typically exhibit higher levels of hormones, particularly during the early fruitsetting stage. For instance, seedless citrus fruits contain four times the IAA content found in seeded varieties [24]. In the present study, GA levels in seedless chestnut rose ovaries progressively increased during the ovarian expansion phase (Fig. 1C). Similar trends had been also observed in pear parthenocarpy, where higher GA levels were detected in parthenocarpic germplasms [15]. In tomato, parthenocarpic lines displayed 3.36 times higher GA concentrations in the early stages of fruit development compared to nonparthenocarpic lines [18]. In the current case, the emasculated flowers were treated with GAs, PAC, and H_2_O. It was demonstrated that GA application not only increased fruitsetting rate, but also promoted fruit expansion and early ripening. In contrast, inhibiting GA biosynthesis resulted in a cessation of fruit weight starting on 7 DAT, with >80% fruit abscission occurring by 30 DAT (Fig. 2). Exogenous application of GAs might also induce parthenocarpy in other species. For instance, apple spraying with 1500 ppm GA_3_ successfully induced parthenocarpy, resulting in seedless fruits [25]. Similarly, GA_3_ application led to the formation of parthenocarpic fruits in apples [26]. In cucumber, treatment with GA_4 + 7_ promoted parthenocarpic fruit formation [27, 28]. Also, spraying GA_4 + 7_ on the pear not only increased the fruitsetting rate compared to pollination, but also produced seedless fruits with higher sugar content [29]. With the endogenous hormone profiling during fruit developmental stages, and exogenous hormone treatments in seedless chestnut rose, GAs were justified to serve as the key hormone regulating the fruitsetting and development, suggesting that GAs play a crucial role in inducing parthenocarpy in this fruit tree.

In the GA biosynthesis pathway, three enzymes play critical roles: GA2ox, GA3ox, and GA20ox. These rate-limiting enzymes directly regulate GA content in organisms, among which, GA3ox and GA20ox are essential biosynthetic enzymes, while GA2ox functions as the critical catabolic enzyme [30]. Genes encoding these three rate-limiting enzymes had been identified in various species. For example, *Arabidopsis* and rice each possess 16 and 21 *GAoxs*, respectively [31], grapevine contains 24 [30], *Lagerstroemia indica* has 36 [32], and apple harbors 41 [33]. In this study, a total of 43 members were identified from seedless chestnut rose, including 14 *RsGA2ox*, 14 *RsGA3ox*, and 15 *RsGA20ox* (Fig. 4A). Functional analyses have revealed species-specific roles for these enzyme families. In pear, *PbGA20ox* enhanced plant height, delayed fruit abscission, and induced parthenocarpy [15]. Potato plants with RNAi *StGA3ox* exhibited the shortened internodes, reduced height, and diminished tuber weight [34]. *Cunninghamia lanceolata* overexpressing *ClGA2ox* shows dwarfism, decreased lignin content, and delayed xylem vessel differentiation [35]. It can be observed that the number of *GAoxs* varies among different species, and each family member may have the potential to induce parthenocarpy.

### *RsGA3ox9* highly contributes to the fruit development of seedless chestnut rose

Currently, heterologous overexpression of *RsGA3ox9* in tomato resulted in taller plants (Fig. S2E). As emasculated before flowering, the WT failed to set fruit, whereas the *RsGA3ox9*-overespressing lines retained the ability to develop fruits normally under emasculated conditions (Fig. 6C). However, this parthenocarpic capability was limited: transgenic plants exhibited only 14.33% fruitsetting rate without pollination (Table 1), possibly due to the restricted function of a single gene (*RsGA3ox9*). Additionally, fruits without seed were also significantly smaller (Table 1), since seed may serve as major sources of hormones in fruits [36]. These endogenous hormones drive ovary development and fruit set [37]. Without the hormones, the division and differentiation of fruit cells will be inhibited, and eventually lead to the cessation in fruit growth and development.

To directly unravel the function of *RsGA3ox9*, we silenced this gene in the rapidly expanding fruits of seedless chestnut rose using VIGS. Silencing efficiency was first validated by detecting significantly reduced *RsGA3ox9* expression on 10 DAT (Fig. 6D). Concurrently, fruits in the experimental group showed the marked size reduction compared to the controls (Fig. 6E), indicating that *RsGA3ox9* positively involves in fruit development. With the increase of the silencing time, fruits were prone to wither and drop on 20 DAT (Fig. 6E). Previous studies had also highlighted the importance of *GA3ox* genes in parthenocarpic plants. For example, pears spraying with 2,4-D increases GA_4_ content and induces parthenocarpic fruits, accompanied by significant upregulation of *PbGA3ox-1* [12]. Similarly, cytokinin-induced parthenocarpic fruits in *Ficus carica* show significant upregulation expression of *FcGA3ox* [38]. Therefore, *RsGA3ox9* plays a critical role in parthenocarpic fruitsetting and development of seedless chestnut rose, which also reflects that seedless chestnut rose may serve as an elite genotype for parthenocarpy breeding in chestnut rose.

### RsMYB8–RsMYB73 module positively regulates *RsGA3ox9* expression

The expression regulation of plant genes involves a complex and precisely orchestrated network, with TFs playing central roles in this process [39]. To gain deeper insights into the regulatory network of *RsGA3ox9*, its *cis*-acting elements revealed the presence of MYB-binding sites (Fig. 4C). Y1H and dual-luciferase reporter assays confirmed that the three TFs, i.e. RsMYB3, RsMYB8, and RsMYB73, bind to the *RsGA3ox9* promoter to regulate transcription (Fig. 7D–H). MYB represents one of the largest families in plants, with 400–500 members identified in a single species [40], that regulate diverse biological processes including root hair development, floral stem strength, pollen formation, fruitsetting and development, and seed germination [41]. MYB also mediates plant responses to abiotic stresses such as drought, high-temperature stress, ultraviolet light, cold stress, and salt stress [42]. CsMYB77 was proved to be involved in citrus fruit size formation and promoted fruit ripening as a negative regulator [24]. SlMYB70 can directly negatively regulate ethylene biosynthesis and affect tomato fruit development [43]. In this study, Y2H and luciferase complementation assays confirmed the interaction between RsMYB8 and RsMYB73, and the RsMYB8-RsMYB73 complex positively regulated *RsGA3ox9* expression (Figs 7E and 8F, G). Although RsMYB3 can regulate the expression of *RsGA3ox9*, no interactions with RsMYB8 as well as with RsMYB73 had been detected. The presumable explanation is that this TF only binds to the promoter to regulate the transcription of *RsGA3ox9* independently, or it may recruit TFs from other families to interact with it and coregulate gene expression. However, these possibilities had not yet been identified, and the full role of RsMYB3 in regulating *RsGA3ox9* remains to be further explored. In addition to coregulating *RsGA3ox9*, MYB8 and MYB73 may recruit other TFs to coregulate *RsGA3ox9*. Furthermore, these TFs might also participate in protein translational modifications (PTMs, e.g. phosphorylation, glycosylation, ubiquitination, methylation, acetylation, etc.) of *RsGA3ox9*, ultimately inducing parthenocarpy. These limitations of the current study include the lack of conclusive evidence to confirm our hypotheses. In subsequent work, we will conduct a more in-depth analysis of the regulatory relationships between them to further clarify the mechanism of parthenocarpy. These findings further highlight the importance of MYB in fruit growth and development, which provides new insights into its regulatory mechanisms since the involvement of MYBs in parthenocarpy has been scarcely reported so far.

**Figure 9:**
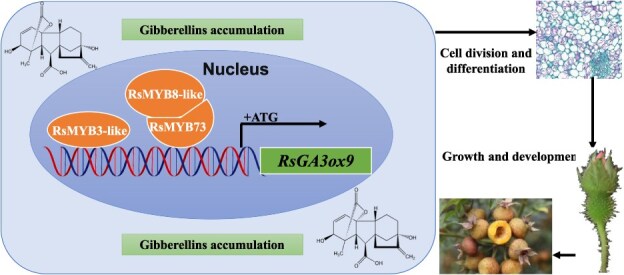
Molecular module of *RsGA3ox9* regulating parthenocarpy in seedless chestnut rose.

Collectively, these findings justified the molecular regulation module of *RsGA3ox9* involved in parthenocarpy of seedless chestnut rose. The TFs of RsMYB3, RsMYB8, and RsMYB73 positively regulate the expression of *RsGA3ox9*, with RsMYB8 and RsMYB73 interacting positively to coregulate *RsGA3ox9*. Meanwhile, the fruit growth and development were promoted by *RsGA3ox9*, and ultimately induced parthenocarpy in seedless chestnut rose (Fig. 9).

## Conclusions

Seedless fruit is a very favorite trait for consumer preference in chestnut rose. In the current study, GA was justified to serve as a key hormone for parthenocarpy induction in seedless chestnut rose, and 43 *RsGAoxs* were identified based on genome-wide analysis. *RsGA3ox9* was justified to substantially involve in parthenocarpy of seedless chestnut rose, and RsMYB8–RsMYB73 complex promotes parthenocarpic fruitsetting by upregulating *RsGA3ox9*. These findings may facilitate the further breeding for seedless fruit in both *R. sterilis* (seedless chestnut rose) and *R. roxburghii* (chestnut rose), and also provide novel insights for better understanding the mechanism underlying the parthenocarpic fruitsetting in fruit species.

## Materials and methods

### Plant materials and growth conditions

In this study, we used seedless chestnut rose, an 8-year-old plant growing in the Agricultural Academy of Anshun, Guizhou Province, China. The site has an altitude of 1395 m, an average annual temperature of 14°C, and an annual rainfall of 1300 mm. The seedless chestnut rose plants were well grown. From April to June 2021, fruits were randomly collected the ovary at the seventh day before flowering (RS1), the ovary at full flowering (RS2), the fruit at the 14th day after flowering (RS3), and the fruit at the 28th day after flowering, each sample was subjected to three biological replicates. All samples are immediately frozen with liquid nitrogen and brought back to the laboratory for storage in a −80°C low-temperature freezer for hormone content determination and transcriptome sequencing.

The transgenic plants used in this study were ‘Micro-Tom’ tomatoes (*Solanum lycopersicum*) [44]. The CaMV35S-*RsGA3ox9* overexpression vector was constructed, and the recombinant vector was transferred to *Agrobacterium tumefaciens* [45], the infection solution was prepared and then infected with tomato cotyledons, which were aseptically mixed and inoculated in screening medium for 3 days. Following the acquisition of resistant seedlings, subculturing was performed every 2 weeks. The seedlings were then transferred to a rooting medium until they reached a specific height and developed roots, after which they were ultimately transplanted into soil for cultivation [46].

### RNA extraction and transcriptome analysis

RNA was extracted from samples using the StarSpin Plant RNA Kit (GenStar, Beijing, China). RNA integrity was assessed via 1% agarose gel electrophoresis, and purity was verified using a Spectrophotometer 1510 (Thermo Fisher Scientific, Finland). Qualified RNA was reverse-transcribed into first-strand cDNA with the PrimeScript™ RT Reagent Kit (Takara, Dalian, China) [47].

The total of 12 samples of cDNA from RS1 to RS4 were sent to Shanghai Majorbio for transcriptomic sequencing. Clean data from all samples were *de novo* assembled using Trinity software. DEGs between groups were analyzed based on TPM values with DESeq2, applying thresholds of |log_2_FC| ≥ 1 and *P*adjust <0.05. Subsequent GO and KEGG enrichment analyses were conducted on the identified DEGs [48]. The data were analyzed on the online tool of Majorbio Cloud Platform (https://cloud.majorbio.com/page/tools/).

### Phytohormone analysis

Endogenous hormone levels were measured in the same samples used for transcriptomic analysis. Fruit tissues were ground into powder and subjected to overnight extraction with acetonitrile. Hormone contents were performed using an Agilent 1290 high performance liquid chromatography (HPLC) system (Agilent Technologies, USA) coupled with a SCIEX 6500 Qtrap tandem mass spectrometer (MS/MS; AB Sciex, USA) [23]. All hormone reference standards were purchased from Sigma-Aldrich (USA). HPLC-grade methanol and acetonitrile were sourced from Merck KGaA (Germany). These quantification experiments were performed by Wuhan ProNets Biotechnology Co, Ltd., Wuhan, China. The experiment was performed with three biological replicates and the raw data is in Table S3.

### Hormone application in plants

Five days before flowering in seedless chestnut rose, nine individual plants with uniform growth were selected. On each plant, 15 similarly sized ovaries were emasculated at five directions (east, west, south, north, center) at the same height (totaling 135 ovaries). These ovaries were then sprayed with ddH_2_O, GA_4 + 7_ (1 mg/ml), and paclobutrazol (1 mg/ml) [25], followed by bagging to prevent unintended pollination.

Fruit set rates under the three treatments were recorded at 7, 14, 30, and 45 DAT. Simultaneously, ovaries were collected at 0, 3, 7, 14, and 21 DAT. One portion of the collected ovaries was flash-frozen in liquid nitrogen, while the other portion was preserved in 60% Formalin-Aceto-Alcohol (FAA) fixative solution (V_formaldehyde_:V_glacial acetic acid_:V_absolute ethanol_ = 18:1:1) and stored at 4°C [49].

### Histological and microscopic examination

Ovaries/fruits sprayed with ddH_2_O, GA_4 + 7_ and Paclobutrazol were fixed in FAA solution. After 3 days, cellular quantity and size were examined. Fixed samples were removed from 60% FAA and processed through gradient dehydration, clearing, and wax infiltration using a dehydration machine (YD-12P, Yidi). Embedding was performed with an embedding machine (YD-6L, Yidi), followed by solidification on a freezing stage (YD-6LA, Yidi). The wax blocks were trimmed and sectioned at 8 μm thickness using a microtome (YD-315, Yidi). Ribbons were mounted on glass slides and baked in a sliding oven (YD-AB2, Yidi). Sections were dewaxed, stained, and dehydrated in a staining machine (YD-700, Yidi), and then mounted for microscopic examination [50]. The scanner (Pannoramic MIDI II, Shanghai Damai Biotechnology Co., Ltd., China) was used for scanning. Software CaseViewer2.3 (https://www.3dhistech.com/solutions/caseviewer/) was used to observe the cellular quantity and size of seedless chestnut rose.

### Gibberellin oxidase gene family identification

The genome data of seedless chestnut rose (https://doi.org/10.1111/tpj.16543) [20] was downloaded for analysis. Candidate genes were initially screened using Hidden Markov Models (HMM) based on conserved domains DIOX_N (PF14226) and 2OG-FeII_Oxy (PF03171) [30, 51]. Subsequently, sequences of the plant GA oxidase gene (*GAox*) family were retrieved from the NCBI Protein database (https://www.ncbi.nlm.nih.gov/protein) and subjected to BLAST homology alignment. The intersection of genes identified by both methods was selected as the final GA oxidase gene set in seedless chestnut rose. Protein sequences of *A. thaliana* (https://www.arabidopsis.org/), *O. sativa* (http://rice.uga.edu/pub/data/Eukaryotic_Projects/o_sativa/annotation_dbs/), *M. domestica* (https://www.rosaceae.org/species/malus/malus_x_domestica/genome_v3.0.a1), and *V. vinifera* (https://evorepro.sbs.ntu.edu.sg/species/view/12) were downloaded. *GAox* sequences were retrieved using published protein IDs and aligned with seedless chestnut rose sequences to construct a phylogenetic tree [52]. All analyses were performed using TBtools-II (https://github.com/CJ-Chen/TBtools-II/releases), with tree visualization conducted on iTOL (https://itol.embl.de/).

### Parthenocarpy capacity of transgenic tomato

To determine parthenocarpic capacity of transgenic tomatoes, six plants per line and four independent lines (WT, OE-2, -7, and -9) were selected, 10–15 flowers were emasculated per plants. Flower emasculation was carried out 2 days before anthesis to prevent self-pollination, and all nonselected flowers were removed. The fruit setting rate and their weights were determined at maturity [18].

### Virus-induced gene silencing in seedless chestnut rose

Specific primers targeting *RsGA3ox9* for gene silencing were designed. A 300-bp fragment of the target gene was amplified by PCR and constructed into the pTRV2 vector. The recombinant plasmid was introduced into *A. tumefaciens* GV3101 via heat shock transformation. For infection, *Agrobacterium* cultures carrying pTRV2-*RsGA3ox9* and pTRV1 were mixed at equal concentrations and volumes. The infection solution contained: 1 ml 0.5 M MES buffer, 0.5 ml 1 M MgCl_2_, 50 μl 1 M acetosyringone (AS), and 50 ml ddH_2_O. After centrifugation, *Agrobacterium* were resuspended to OD_600_ = 0.6 and incubated for 3 h before application.

In the late afternoon, uniformly sized fruits during the rapid growth period were selected. Using a syringe, collect the infection fluid and inject it into the fruit flesh. Cover the fruits with paper bags to prevent light exposure. The experimental group received a mixture of pTRV2-*RsGA3ox9* and pTRV1, while the control group received pTRV2 and pTRV1 [53]. After 10 days, the fruit sizes and gene expression levels were measured in both groups.

### Yeast one-hybrid assay

The 1000-bp sequence of the gene *RsGA3ox9* promoter was constructed into the pHIS2 vector, the complete CDS of the genes RsMYB3, RsMYB8, and RsMYB73 were constructed into the vector pGADT7. The recombinant plasmids pHIS2-*pro RsGA3ox9* and pGADT7-RsMYB3/RsMYB8/RsMYB73 were cotransferred into Y187 yeast strain. All of them were incubated on the SD-Trp-Leu solid medium (Coolaber, Beijing, China) at 30°C for 2 days. After PCR confirmation as positive colonies, single colonies were individually transferred into ddH_2_O with OD_600_ = 0.05. Subsequently, 1.5 μl of the bacterial suspension was spotted onto the SD-Trp-Leu-His solid culture medium containing 0, 15, 30, 45, and 60 mM of 3-AT concentration. The results were observed after 3 days of incubation in a 30°C incubator [54].

### Dual-luciferase and luciferase complementation assay

pGreenII 0800-LUC-*pro RsGA3ox9* and pGreenII 62-SK-RsMYB3/RsMYB8/RsMYB73 vectors were constructed. These recombinant plasmids were introduced into *A. tumefaciens* GV3101 and infiltrated into tobacco leaves. After 2 days of dark incubation, luciferase expression was observed using the PlantView600 *in vivo* imaging system (BLT, Guangzhou, China). Simultaneously, infiltrated leaf tissues were ground to extract luciferase, and dual-luciferase activity was measured with a Spectrophotometer 3001 (Thermo Fisher Scientific, Finland). Similarly, the pCambia1300-nLuc-RsMYB8 and pCambia1300-cLuc-RsMYB73 recombinant vectors were constructed, transformed into GV3101, and their expression patterns in tobacco were further analyzed [55].

### Yeast two-hybrid assay

The CDS of transcription factors RsMYB3, RsMYB8, and RsMYB73 were individually cloned into the Y2H vectors pGADT7 and pGBKT7. The recombinant plasmids pGBKT7-RsMYB3, pGBKT7-RsMYB8, and pGBKT7-RsMYB73 were first transformed into yeast strain Y2H. Transformed yeast cells were plated on SD-Trp solid medium (Coolaber, Beijing, China) and incubated at 30°C for 2 days. After the positive strains were identified by PCR, single colonies were respectively picked and suspended in ddH_2_O to make the OD_600_ = 0.05. Subsequently, 1.5 μl of the bacterial suspension was pipetted and placed on SD-Trp-His-Ade solid medium (Coolaber, Beijing, China) coated with 4 mg/ml X-α-gal. First, the self-activation was observed. If there is no self-activation, the combinations of pGADT7-RsMYB3/RsMYB8/RsMYB73 and pGBKT7-RsMYB3/RsMYB8/RsMYB73 were respectively cotransformed into the yeast strain. The positive single colonies were spotted onto the SD-Trp-Leu-His-Ade solid medium (Coolaber, Beijing, China) coated with 4 mg/ml X-α-gal. The medium was then placed in an incubator at 30°C. The results were observed after 3 days of cultivation [56].

### Subcellular localization analysis

After removing the stop codons from the CDS of the gene *RsGA3ox9* and the transcription factors RsMYB3, RsMYB8, and RsMYB73, they were constructed into the vector pCambia1300-35S-EGFP. The recombinant plasmids were transformed into *A. tumefaciens* strain GV3101. An infiltration solution was prepared (1 ml of 0.5 M MES + 0.5 ml of 1 M MgCl_2_ + 50 μl of 1 M AS + 50 ml of H_2_O), and its OD_600_ was adjusted to 1. The solution was incubated in an incubator at 30°C for 3 h and then injected into tobacco leaves. After 2 days of dark cultivation, the localization was observed using a confocal laser scanning microscope (TCS SP8, Weztlar, Germany) [57].

### Quantitative real-time PCR

For seedless chestnut rose and tomato samples, total RNA was extracted and reverse-transcribed into cDNA. qPCR was performed using primers for reference genes and target genes. Data were analyzed with Student’s *t-*test, and relative expression levels were calculated using the 2^−ΔΔCt^ method [58]. Three biological replicates and three technical replicates were performed on each sample. The primer sequences of qRT-PCR are provided in Table S4.

### Statistical analysis

All data were analyzed for statistical significance using IBM SPSS Statistics 26 (https://www.ibm.com/spss). Distinct letters represent statistically significant variances at a significance level of *P* < 0.05. Data visualization was performed using Origin 2021 (https://www.originlab.com/).

## Supplementary Material

Web_Material_uhaf277
