# Supplementary material for: RsMYB8-RsMYB73 module positively regulates parthenocarpic fruitsetting via elevating RsGA3ox9 expression in seedless chestnut rose (Rosa sterilis)
Source: Hortic Res. 2025 Oct 20;13(1):uhaf277. doi: 10.1093/hr/uhaf277 (PMC12881858; doi:10.1093/hr/uhaf277)
Supplement: Web_Material_uhaf277 [file web_material_uhaf277.zip › Supplementary Figures.docx]

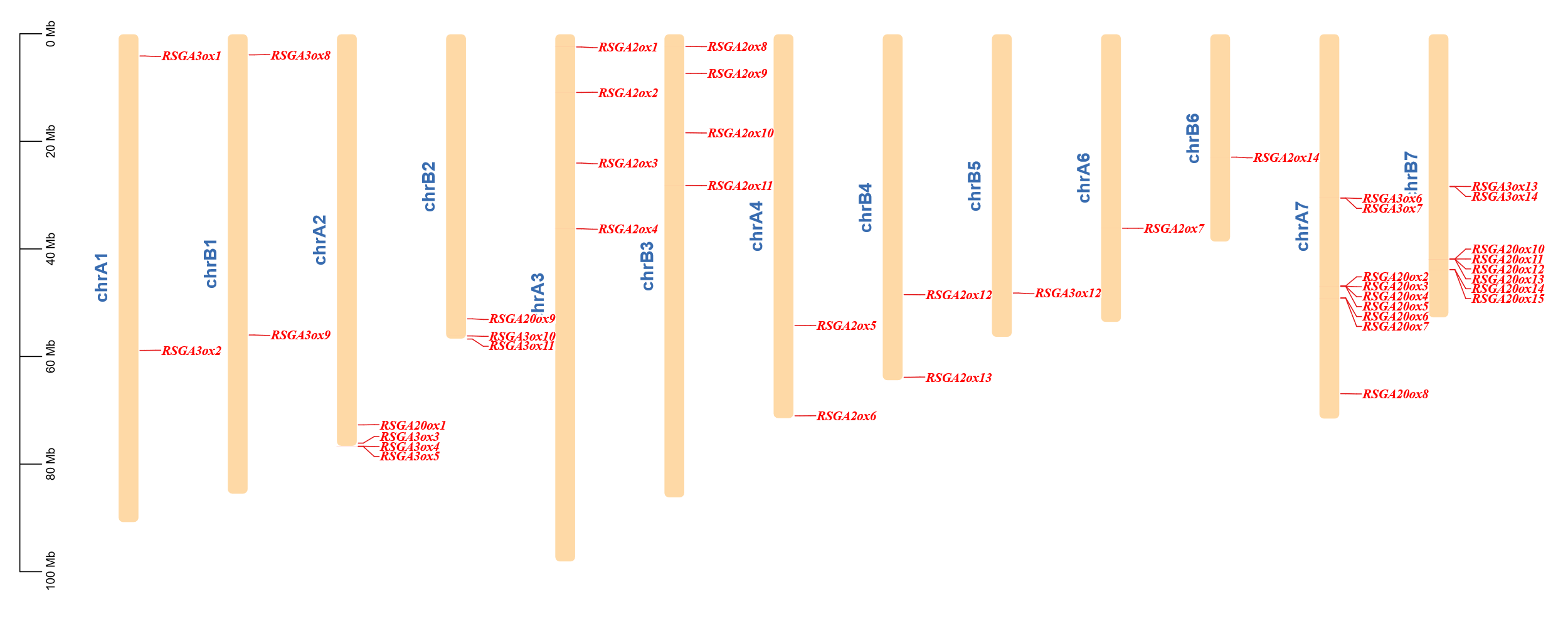


**Figure S1 Chromosomal localization of 43 *RsGAoxs***


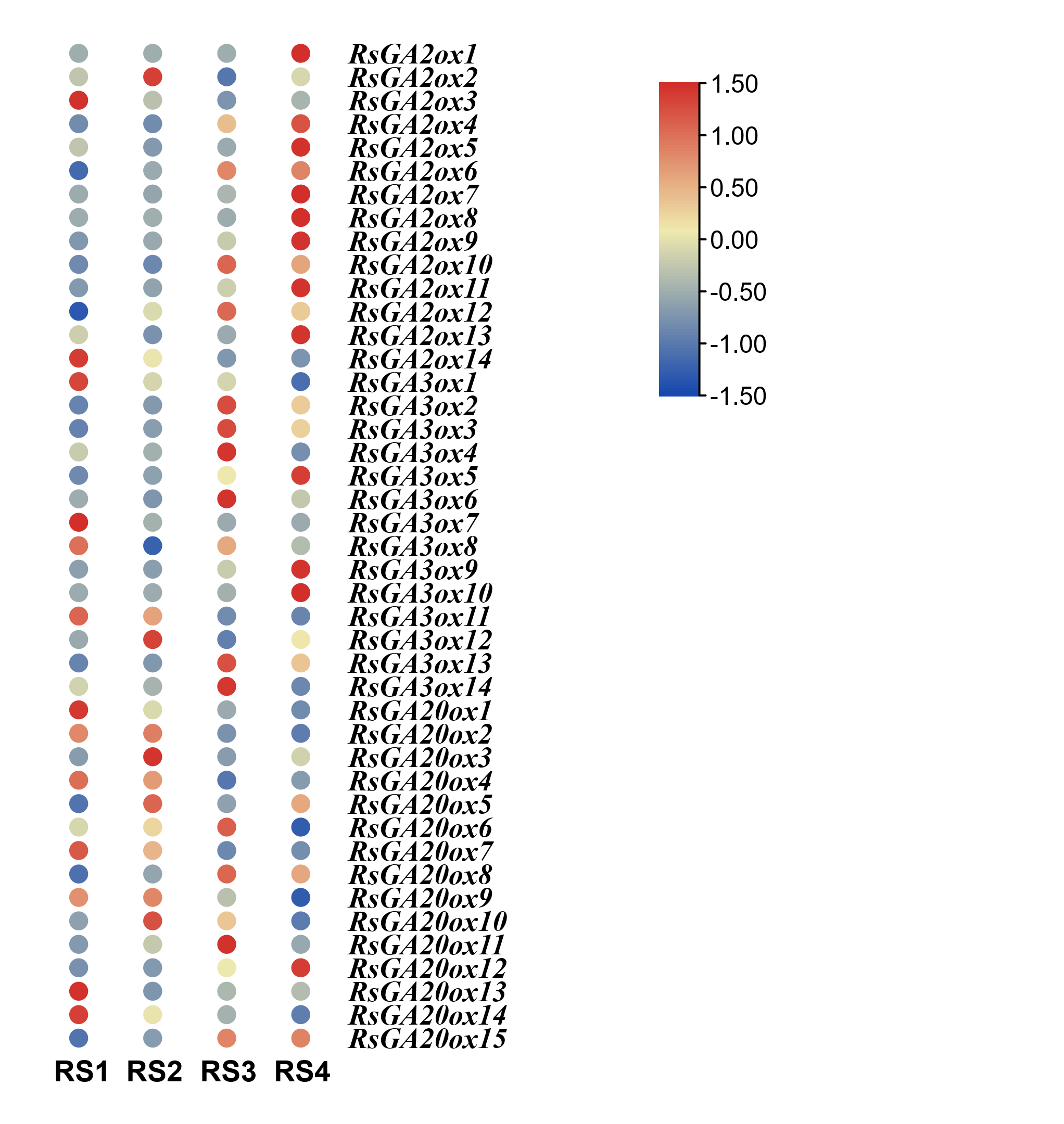


**Figure S2 The TPM (Transcripts Per Million) heatmap of 43 *RsGAoxs***


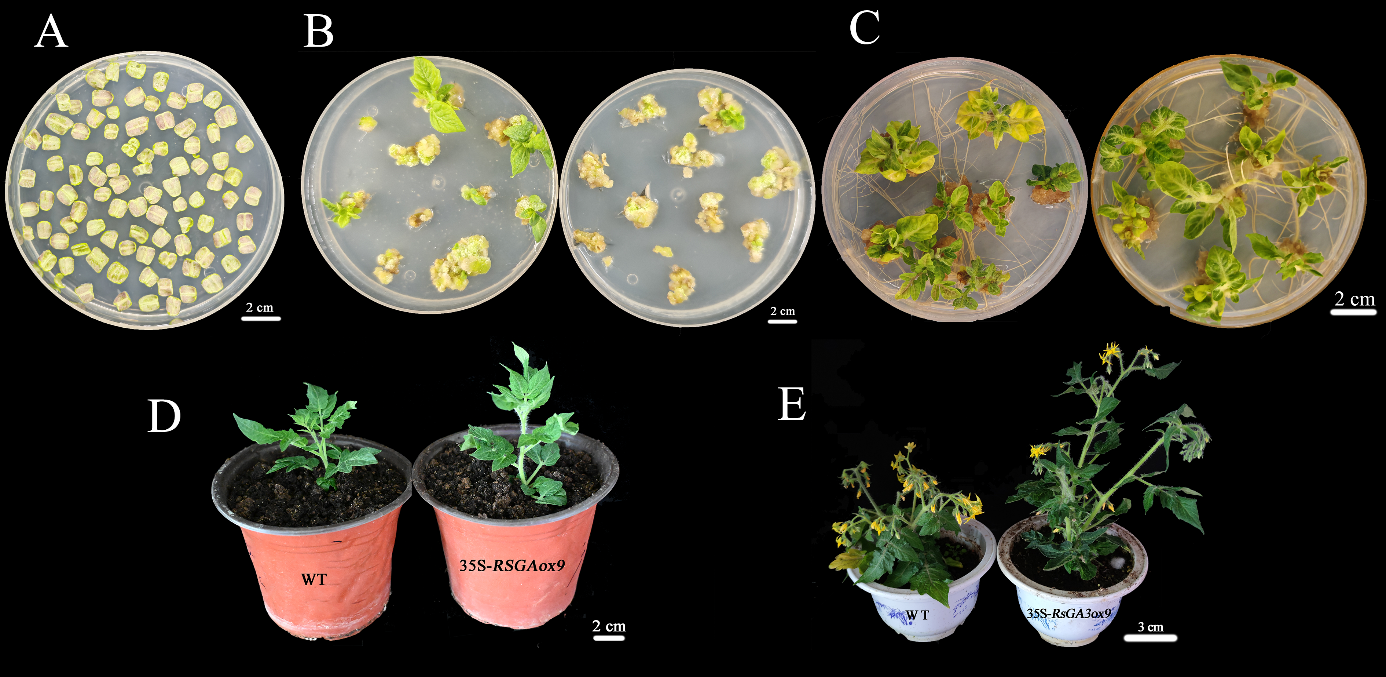


**Figure S3 The overexpression lines of *RsGA3ox9* in tomato**

(A) Selection process of overexpressing tomato plants. (B) Differentiation process of overexpressing tomato plants. (C) Rooting process of overexpressing tomato plants. (D) Seedlings of WT and overexpressing tomato plants. (E) Mature WT and overexpressing tomato plants.


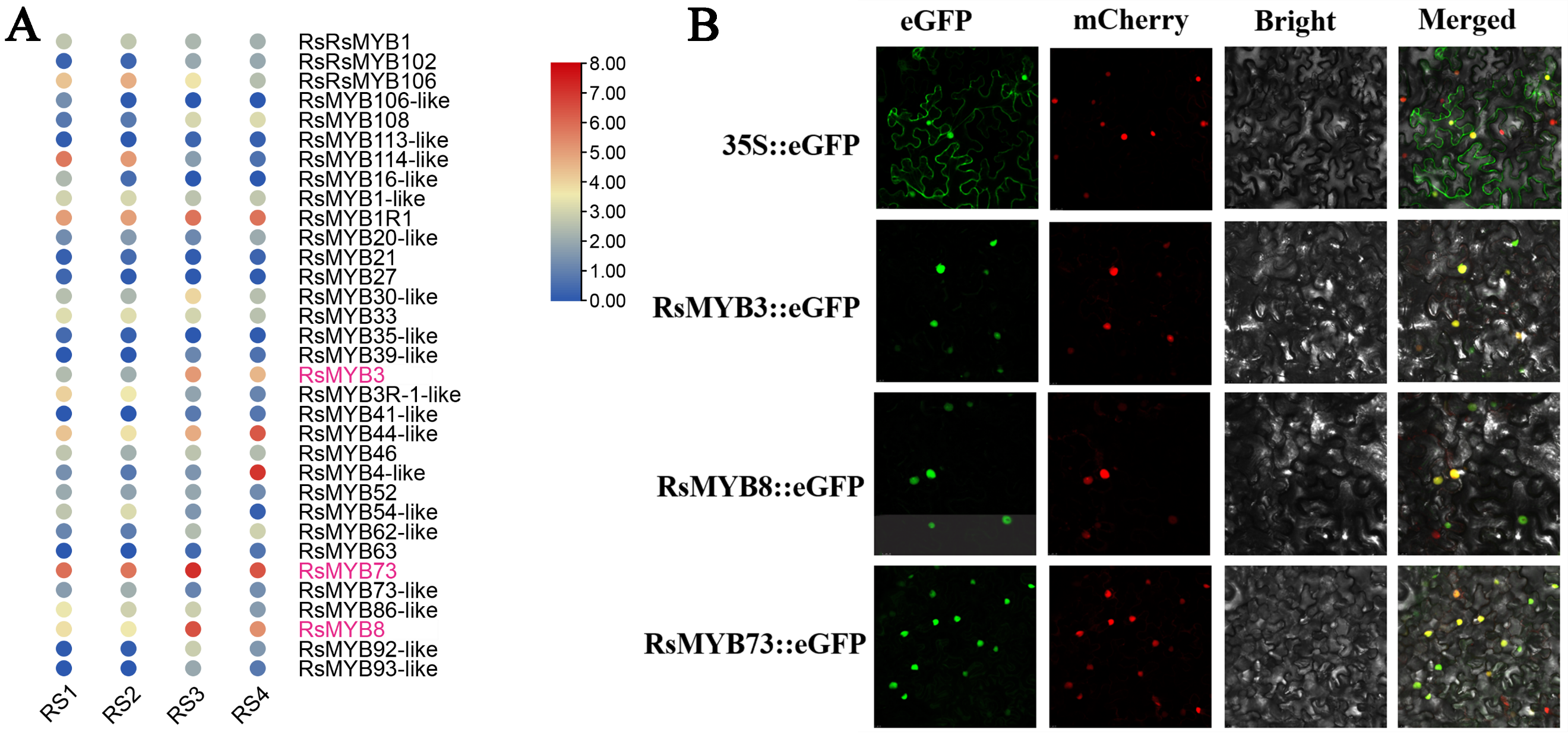


**Figure S4 TPM heatmap and subcellular localization of RsMYBs**

(A) The TPM of RsMYBs across four developmental stages in transcriptome. (B) Subcellular localization of RsMYB3, RsMYB8 and RsMYB73 in tobacco leaves.


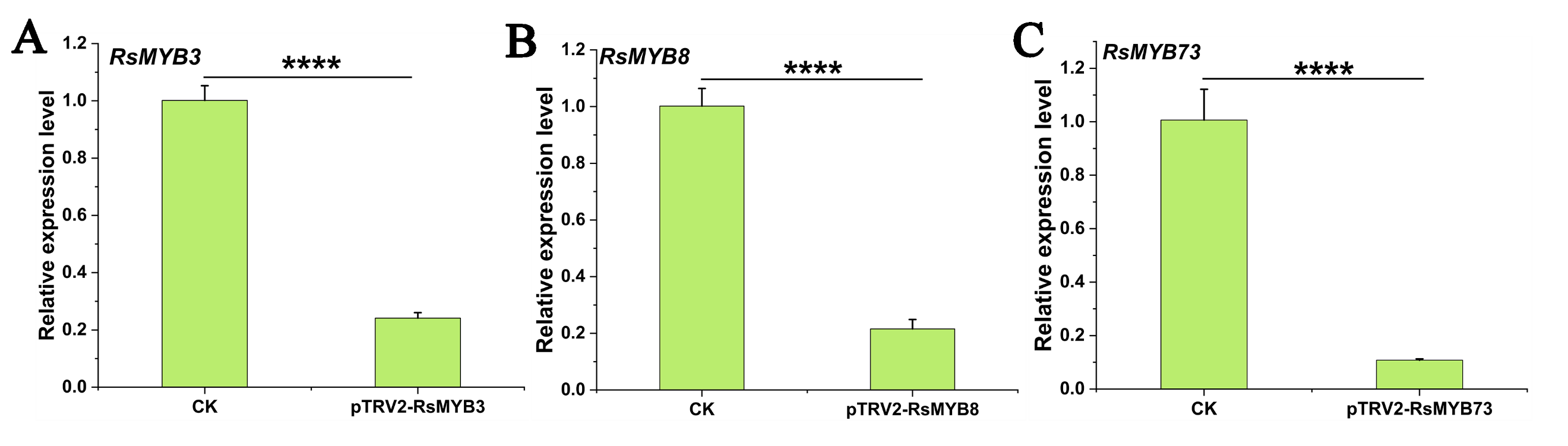


**Figure S5 The** **relative expression level of RsMYBs by VIGS in** **seedless chestnut rose fruit**

(A) Relative expression level of RsMYB3. (B) Relative expression level of RsMYB8. (C) Relative expression level of RsMYB73. Data with three biological replicates were given as mean values ± SD, asterisk indicates significant difference (Student’s *t*-test, *****P* < 0.0001).
